# Higher Mean Arterial Pressure during Cardiopulmonary Bypass May Not Prevent Acute Kidney Injury in Elderly Patients Undergoing Cardiac Surgery

**DOI:** 10.1155/2022/7701947

**Published:** 2022-04-14

**Authors:** Yunfen Ge, Tapas Ranjan Behera, Ming Yu, Shuyang Xie, Yue Chen, Hui Mao, Qiong Xu, Yu Zhao, Shuijun Zhang, Quanquan Shen

**Affiliations:** ^1^Center for Rehabilitation Medicine, Department of Anesthesiology, Zhejiang Provincial People's Hospital (Affiliated People's Hospital, Hangzhou Medical College), Hangzhou, Zhejiang 310014, China; ^2^Taussig Cancer Institute, Cleveland Clinic, Cleveland, OH 44195, USA; ^3^Department of Anesthesiology, Tiantai People's Hospital, Tiantai, Taizhou, Zhejiang 317299, China; ^4^Geriatric Medicine Center, Department of Endocrinology, Zhejiang Provincial People's Hospital (Affiliated People's Hospital, Hangzhou Medical College), Hangzhou, Zhejiang 310014, China; ^5^Center for Plastic & Reconstructive Surgery, Department of Orthopedics, Zhejiang Provincial People's Hospital (Affiliated People's Hospital, Hangzhou Medical College), Hangzhou, Zhejiang 310014, China; ^6^Urology & Nephrology Center, Department of Nephrology, Zhejiang Provincial People's Hospital (Affiliated People's Hospital, Hangzhou Medical College), Hangzhou, Zhejiang 310014, China

## Abstract

We aimed to evaluate the role of higher mean arterial pressure (MAP) during cardiopulmonary bypass (CPB) in preventing development of acute kidney injury (AKI). *Methods*. We evaluated a population of elderly individuals >60 years of age undergoing CPB to find correlation of MAP during CPB with development of AKI after the surgery. Patients who experienced sustained low MAP during the CPB defined as that of <65 mmHg were compared with those that had sustained high MAP of >65 mmHg for their outcome with regard to AKI. The KDIGO criteria were used to define presence of acute kidney injury. *Results*. Of the total 92 patients, 50 were in the low-pressure group and 42 were in the high-pressure group. The MAP was 61.14 ± 5.54 mmHg in the low-pressure group and 68.97 ± 3.65 mmHg in the high-pressure group (*p* < 0.001). 13 (26%) in the low-pressure group and 17 (40.48%) in the high-pressure group developed AKI (*p* = 0.140). Male sex was associated with an increased incidence of cardiac surgery-associated AKI (*p* = 0.034). *Conclusions*. A higher MAP in the range of 65–75 mmHg during the cardiopulmonary bypass does not significantly prevent acute kidney injury in elderly patients undergoing cardiac valve surgery.

## 1. Introduction

Acute kidney injury (AKI) is a known complication of cardiac surgery. It is the strongest risk factor of end-stage renal disease and mortality in patients undergoing cardiac surgery [1, 2]. Although overall morbidity and mortality after cardiac surgery have decreased in the past decade, the incidence of AKI after cardiac surgery has not changed [3, 4]. Effective prevention and management of postoperative AKI need better understanding of the pathophysiology. The etiology behind the development of AKI after cardiac surgery is likely multifactorial and is incompletely understood [5, 6]. Activation of proinflammatory mediators, direct nephrotoxicity, reduced renal perfusion pressure, hemolysis, severe hemodilution during cardiopulmonary bypass (CPB), and low oxygen delivery (DO_2_) [7, 8] are thought to be the main factors contributing to AKI. CPB is necessary to facilitate a motionless and bloodless surgical field for cardiac surgery. However, during the CPB, contact of blood components with the artificial surface of the bypass circuit can result in significant inflammation and oxidant stress response contributing to end organ damage [9–11]. Furthermore, CPB itself decreases the effective renal perfusion pressure up to 30% by altering the vasomotor tone and exposes the renal parenchyma to reduced oxygen tension. The range of renal autoregulation has been found to be between 75 mmHg and 160 mmHg in an experimental setting [12]. The arterial pressure is below this range in the majority of the time during CPB [13]. It is generally maintained at 40–60 mmHg. Furthermore, the pressure during CPB is continuous and not pulsatile as under normal conditions [14]. We hypothesized that a higher-than-spontaneous mean arterial pressure (MAP) during CPB with the intent of bringing the arterial pressure close to the renal autoregulatory range may improve microcirculation and thus decrease the risk of AKI after cardiac surgery.

## 2. Patients and Methods

### 2.1. Participants

Patients who underwent cardiac valve surgery at our quaternary academic medical center from May 1, 2019, to December 31, 2020, were evaluated through their medical records for eligibility for inclusion in this retrospective study. The study followed the international regulations in accordance with the Declaration of Helsinki. The study protocol was approved by the Medical Ethics Committee of Zhejiang Provincial People's Hospital (no. 2018KY034). Written informed consent was obtained from all eligible patients prior to their enrollment in the study. All operations were performed by the identical surgery team.

### 2.2. Inclusion and Exclusion Criteria

All patients who were of age >60 years and had cardiac valve surgery with a CPB between May 1, 2019, and December 31, 2020, were evaluated by retrospective chart review. Inclusion criteria were duration of CPB at least of 90 minutes and MAP during the CPB sustained either below or above 65 mmHg without crossing the 65 mmHg pressure threshold for a prolonged time. Exclusion criteria were presurgical evaluation of serum creatinine (sCr) > 200 *μ*mol/L or estimated glomerular filtration rate (eGFR) < 30 mL/min/1.73 m^2^, previous heart surgery, endocarditis, liver dysfunction, dialysis, AKI, total phenylephrine intravenous doses >2.0 mg and/or continuous intravenous infusion of norepinephrine (NE) > 0.1 *μ*g/kg/min in the operation, organ transplantation, or any case of an acute cardiac surgery after myocardial infarction, defined as a coronary angiography in <24 h of surgery. Patients who had sustained MAP in the range of 50 mmHg to 65 mmHg were categorized as the low-pressure group (LPG), and those who had sustained MAP in the range of 65 mmHg to 80 mmHg during the CPB were defined to belong to the high-pressure group (HPG). Patients reported to have had fluctuation in MAP ranging between both groups were excluded from the evaluation.

### 2.3. Cardiopulmonary Bypass

All patients were premedicated with midazolam 0.1 mg/kg orally the night prior to surgery. Anesthesia was induced with fentanyl (10* μ*g/kg), propofol (1–2 mg/kg), and cisatracurium (0.2 mg/kg). Maintenance of anesthesia was achieved using continuous infusion of remifentanil (10–20 *μ*g/kg/hour) and propofol (3–8 mg/kg/hour).

CPB was performed after systemic heparinization 300 U/kg and maintaining an activated clotting time (ACT) longer than 480s, and temperature-adjusted flow rates were 2.4 L/(min·m^2^) at the time of CPB. The priming volume of the extracorporeal circuit was about 1500 ml, which was adjusted according to the patients' body weight, and constituted ringer's solution 1200 ml, 20% albumin 100 ml, 20% mannitol 150 ml, and 5% sodium bicarbonate 50 ml. After CPB was established, cooling was initiated, and the patient's temperature was allowed to drift to 34°C. After clamping of the ascending aorta, cardiac arrest was achieved with cold cardioplegic solution. Cardioplegia was prepared by our perfusionist, which is mixed with blood in a ratio of one part crystalloid Del Nido cardioplegia to four part fully oxygenated patient's whole blood [15, 16]. The modified cardioplegia can decrease the extent of hemodilution, and the entire volume enters the primary circulation. During CPB, goal-directed perfusion is managed to maintain the DO_2_ level above the identified critical value of 272 mL/min/m^2^ [7, 17]. Due to unavailability of dedicated software system to collect metabolic parameters, DO_2_ and carbon dioxide production (VCO_2_), the blood gas analysis was performed every half hour, and DO_2_ was calculated using the following equation: DO_2_(mL/min/m^2^) = 10  × pump flow (L/min/m^2^) ×  arterial O_2_ content (mL/100 mL), where arterial O_2_ content was calculated as follows: arterial O_2_ content (mL/100 mL) = hemoglobin (mg/dL) × 1.34 ×  hemoglobin saturation (%) + 0.003 × O_2_ tension (mmHg) [7]. We paid close attention to the hematocrit, which was sustained at around 25%. If the hematocrit was below 25%, we increased the perfusion flow or used ultrafiltration to adjust the perfusion strategy, and red blood cells (RBCs) were transfused when the hematocrit remained still below 25%.

MAP was recorded through a cannula placed in the radial artery. The MAP during CPB was sampled electronically every five minutes by the heart-lung machine. The average MAP during CPB was calculated using the values from on pump to off pump event and was documented.

In all patients, an intravenous bolus of 10 mg of dexamethasone each was given at the starting of CPB, and an intravenous bolus of 20 mg of furosemide was given just before off pump. In cases where the hematocrit was <25%, 1 unit of RBCs was transfused on an as-needed basis.

### 2.4. Postoperative Evaluation

Patients were transferred to cardiac intensive care unit (ICU) for further monitoring and recovery, where they were monitored to maintain adequate cerebral, cardiovascular, pulmonary, and renal function under the meticulous observation of one intensive and critical care resident and fellow each per shift. Patients were discharged from ICU once they were conscious and hemodynamically stable with spontaneous ventilation, without any active bleeding or life-threatening arrhythmias.

### 2.5. Data Collection

Trained staff collected detailed data from recruited patients from the electronic medical records at our medical center. Patients' clinical characteristics collected for each patient included age, sex, height, weight, body mass index (BMI, calculated based on height and weight recorded by the nurse on the day of hospital admission), diabetes mellitus, hypertension, chronic obstructive pulmonary disease (COPD), preoperative hemoglobin, hematocrit, preoperative serum creatinine (sCr), left ventricular ejection fraction (LVEF), and EuroSCORE II. Intraoperative data included operative time, CPB time, aortic cross-clamp time, amount of intraoperative fluid, plasma and red blood cell infusion, and the type of surgery (mitral valve replacement (MVR), aortic valve replacement (AVR), or tricuspid valvuloplasty (TVP)). Intraoperative hemodynamic parameters included mean arterial pressure (MAP) and the dosage of NE and phenylephrine infusion; postoperative data included hemoglobin, hematocrit, LVEF, extubation time, length of ICU, and postoperative hospital stay.

The sCr measurement in the preanesthetic checkup was used as the baseline preoperative value. Preoperative estimated glomerular filtration rate was calculated based on sCr using the Modification of Diet in Renal Disease (MDRD) formula. The intraoperative data including the type of surgery, operative notes, range of sustained MAP, CPB duration, cross-clamp time, blood product transfusion, and fluid requirement, along with the postoperative serum creatinine concentrations measured each morning until the discharge of the patient from the hospital were obtained by reviewing the patient medical records. Other outcome measures collected were the in-hospital mortality, renal replacement therapy, duration of ventilation, and length of stay in the ICU and hospital.

AKI was defined according to the Kidney Disease Improving Global Outcomes (KDIGO) criteria as follows: an absolute increase of sCr 0.3 mg/dL within 48 hours or relative change higher than 1.5-fold from baseline within 7 days or urine output less than 0.5 mL/kg/h for at least 6 hours [18].

### 2.6. Statistical Analyses

The statistical analyses were performed using the SPSS statistical package, version 16.0 (SPSS Inc., Chicago, IL, USA) for Windows. Data are presented as mean ± standard deviation, and frequencies and percentages were used for categorical variables. Student's *t*-test was used to compare the means of normally distributed continuous variables, and the Mann–Whitney *U* test was used for continuous variables that were not normally distributed. Differences among categorical variables were analyzed using Pearson's chi-squared test. A *p* value of <0.05 was considered statistically significant.

## 3. Results

A total of 92 eligible patients undergoing valve surgery with CPB who had sustained low or high MAP during the CPB were analyzed which included 42 in the high-pressure group (HPG) and 50 in the low-pressure group (LPG).

No differences in preoperative data were found between the groups (Table 1).

Intraoperative data were comparable between the groups except the MAP, which was 61.14 ± 5.54 mmHg and 68.97 ± 3.65 mmHg in the LPG and HPG, respectively (*p* < 0.001) (Table 2), and the dose of NE during CPB in the HPG was 8.71 ± 10.07 *μ*g/kg.

Since intravenous bolus of furosemide (20 mg) was given just before off pump, the AKI diagnostic basis of urine volume is not credible, so the diagnosis of AKI was determined based on changes in creatinine; 30 (32.61%) of the patients developed AKI according to the KDIGO criteria. No significant differences were found in the incidence of AKI between the groups which was 17 (40.48%) and 13 (26%) in the HPG and LPG, respectively (*p* = 0.140). sCr concentrations expectedly peaked on the first day after surgery and then gradually declined in most patients. Among the patients with AKI, most of the sCr (27/30) elevations were in KDIGO AKI stage 1, and only two patients in HPG and one patient in LPG had developed creatinine more than 2 times the preoperative level (Table 3).

**Figure 1:**
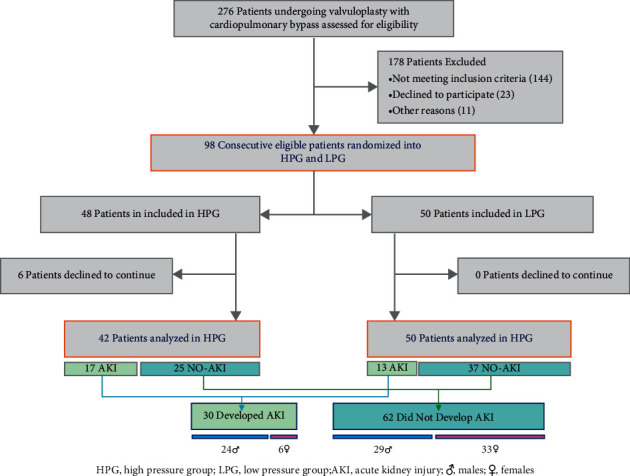
Patient flow diagram.

The results of a subanalysis comparing patients who developed AKI (AKI) with patients who did not develop AKI (NO-AKI) looking at specific risk factors are presented in Table 4; male sex was associated with an increased incidence of cardiac surgery-associated AKI (*p* = 0.034) (Table 4). A participant flow diagram is presented in Figure 1.

## 4. Discussion

This is the first study to investigate the relationship between an intended higher pressure during CPB and development of AKI after elective valve surgery in elderly patients. In previous studies, the presence of older age, hypertension, chronic obstructive pulmonary disease, and diabetes was found to be an independent predictor for development of AKI after cardiac surgery [19–21]. We present a study conducted on cardiac surgery patients who had a high risk of developing AKI by having age >60 years and complex cardiac procedures as inclusion criteria. The primary finding of this study was that a sustained higher MAP of >65 mmHg achieved by higher CPB flow and norepinephrine did not result in any significant differences in incidence of AKI when compared with a spontaneous lower intraoperative arterial pressure. The study population had an AKI incidence of 32.61% in total, the rates of AKI in our study were lower, and the rate among patients undergoing heart valve surgery in previous observational studies was 38.8% [22]. Among the patients developing AKI, most of the serum creatinine elevations were in KDIGO stage 1, and only three patients had creatinine levels more than 2 times the preoperative level. The intraoperative use of intravenous bolus of dexamethasone, furosemide, and restrictive red blood cell transfusion may reduce the severity of the AKI [23–25]. The subgroup analysis resulted in no difference between the duration of ventilation, length of ICU stay, and length of postoperative hospital stay between the AKI and NO-AKI patients.

The mechanisms of CPB-induced AKI include renal hypoperfusion from low-flow, low-pressure, non-pulsatile perfusion with hemodilution and hypothermia, as well as inflammatory response that may induce afferent arteriolar constriction [18]. Lee et al. developed cardiac-renal perfusion models, where they simulated three potential strategies for maintaining optimal renal perfusion during CPB and tested their effectiveness. The strategies were (1) increasing the pump flow; (2) administrating noradrenaline (vasopressor); and (3) administrating fenoldopam (renal vasodilator) [26]. Simulations have revealed that administration of fenoldopam is likely to be the most effective strategy of the three strategies. Another finding from their simulations was that renal autoregulation is likely inoperative during CPB, as evidenced by an unchanging renal vascular resistance with increasing cardiac output and blood pressure. The absolute difference in MAP levels between groups (7.83 mmHg) was considerable, supporting the clinical relevance of the intervention conducted. NE increases blood pressure while constricting renal artery, which has limited improvement in increasing renal perfusion. Obviously, the administration of exogenous NE is effective to support the MAP during CPB; however, in contrast to these benefits, prolonged adrenergic stress is detrimental to the renal artery system. In this study, we excluded some patients with continuous intravenous infusion of NE > 0.1 *μ*g/kg/min during CPB to avoid the adverse effects of NE.

A recent study with a more direct approach of measuring renal function by renal vein catheterization suggests that a higher flow than 2.4 L/min/m^2^ is beneficial for the kidneys. The increased flow rates used were 2.7 L/min/m^2^ and 3.0 L/min/m^2^ which improved the renal oxygen supply/demand relationship by 14 and 30%, respectively [27]. In this study, the patients experiencing sustained higher AP during the CPB were maintained by modulating the flow of the heart-lung machine up to a maximum flow of 110% followed by the use of norepinephrine when needed. Norepinephrine causes intrarenal blood flow redistribution, preserving the cortical perfusion and oxygenation while reducing medullary perfusion. Our current findings provide no support for the proposition that renal perfusion is better during CPB under high pressure induced by norepinephrine. Lankadeva et al. found that medullary perfusion and tissue oxygen tension (PO_2_) could be maintained at low-dose metaraminol, an *α*1-adrenoceptor agonist, because low-dose metaraminol increased the perfusion pressure without affecting renal vascular resistance [28].

In a study by Skytte et al. involving patients undergoing liver transplantation, data on renal blood flow (RBF) and glomerular filtration rate (GFR) were obtained by the renal vein thermodilution technique and renal extraction of chromium ethylenediaminetetraacetic acid (51Cr-EDTA), and it was found that RBF and GFR are pressure-dependent at mean arterial pressure (MAP) levels below 75 mm Hg [29]. At a target MAP of 75 mm Hg, RBF (18%) and GFR (31%) were higher compared to a target MAP of 60 mm Hg. Increasing MAP from 75 up to 90 mm Hg had no further effects on RBF or GFR. Their results suggest that MAP should probably be targeted to be approximately 75 mm Hg for optimal perioperative renal filtration, perfusion, and oxygenation in patients undergoing liver transplantation [29]. In our study, the MAP during CPB was maximally from 65 up to 75 mmHg in the high-pressure group which would have helped to prevent cerebral hyperperfusion.

We found that males were more likely to develop AKI than females. Our data are similar to those previously reported from the United States where Hsu et al. utilized the National Inpatient Sample to identify patients with dialysis-requiring AKI among 24 million hospitalizations, constituting a nationally representative sample of all hospitalizations in the United States between the years 2007 and 2009, and men were 1.9 times more likely to develop AKI than women [30].

CPB time did not influence the development of postoperative AKI. The AKI group did not have significantly longer duration of CPB than NO-AKI group; this result was not consistent with findings by Xu et al. [31], and they studied 115 patients who underwent emergency thoracic aortic operation. The longer duration and lower temperature of CPB in their study were independently associated with an increased hazard of AKI development after thoracic aortic surgery for acute DeBakey Type I aortic dissection.

Our study had several limitations. First, this study used an MAP threshold of 65 mmHg in subdividing the groups with the hypothesis that the patients in the high-pressure group have increased renal perfusion; however, because renal autoregulation is likely inoperative during CPB, as evidenced by an unchanging renal vascular resistance with increasing cardiac output and blood pressure, a higher MAP may not be truly corresponding to a sufficient increase in renal perfusion to influence the development of AKI. Second, the KDIGO criteria used to define AKI are based on sCr, which vary depending on age, gender, muscle mass, and race; the sCr in the postoperative period can also vary considerably depending on the degree of hemodilution, making the values less reliable. Third, due to retrospective nature of the study, there were no data to indicate the measure of tissue perfusion and oxygenation. Specific techniques such as renal artery flow probe, fiberoptic probes, and the probe in the bladder catheter equipped to measure urinary oxygenation could have indicated the contribution of increased AP in improving renal perfusion during CPB and its role thereof in preventing AKI. Further investigations are needed to evaluate the role of other hypothesized contributors of AKI in elderly patients undergoing cardiac surgery.

In summary, a higher mean arterial pressure of >65 mmHg during cardiopulmonary bypass did not decrease the incidence of acute kidney injury after cardiac surgery. Male sex is associated with an increased incidence of cardiac surgery-associated AKI.

## Figures and Tables

**Table 1:** Characteristics of the study patients at baseline.

	HPG (*n* = 42)	LPG (*n* = 50)	*p* value	95% CI
Age (years)	70.52 ± 4.79	70.44 ± 4.70	0.933	−1.89 to 2.06
BMI (kg/m^2^)	23.10 ± 3.98	23.16 ± 3.18	0.932	−1.55 to 1.42
Male, *n* (%)	26 (62%)	27 (54%)	0.364	
Hypertension, *n* (%)	15 (36%)	23 (46%)	0.318	
DM, *n* (%)	3 (7%)	8 (16%)	0.192	
COPD, *n* (%)	3 (7%)	5 (10%)	0.628	
Hemoglobin (g/L)	127 ± 16.45	126.28 ± 17.28	0.839	−6.31 to 7.75
Hct (%)	38.42 ± 4.69	38.66 ± 5.08	0.818	−2.28 to 1.80
Preoperative sCr (*μ*mol/L)	83.97 ± 16.30	80.31 ± 15.96	0.282	−3.05 to 10.35
eGFR mL/ (min·1.73 m^2^)	80.62 ± 16.52	85.04 ± 18.49	0.234	−11.75 to 2.91
Preoperative LVEF (%)	58.98 ± 8.17	60.40 ± 8.28	0.411	−4.85 to 2.00
EuroSCORE II	3.50 ± 1.25	3.64 ± 1.17	0.582	−0.64 to 0.364

Percentages are given as total within group; continuous data are presented as mean ± standard deviation; HPG, high-pressure group; LPG, low-pressure group; CI, confidence interval; BMI, body mass index; DM, diabetes mellitus; COPD, chronic obstructive pulmonary disease; Hct, hematocrit; sCr, serum creatinine; eGFR, estimated glomerular filtration rate; LVEF, left ventricular ejection fraction.

**Table 2:** Intraoperative data.

	HPG (*n* = 42)	LPG (*n* = 50)	*p* value	95% CI
Aortic cross-clamp time (min)	105.33 ± 36.21	100.92 ± 45.14	0.611	−12.77 to 21.59
CPB time (min)	145.45 ± 48.55	142.12 ± 54.62	0.760	−18.27 to 24.93
Operation time (min)	268.70 ± 100.32	303.04 ± 83.88	0.077	−72.49 to 3.80
Postoperative LVEF (%)	56.22 ± 7.25	57.28 ± 7.59	0.501	−4.18 to 2.06
AP (mmHg)	68.97 ± 3.65	61.14 ± 5.54	**<0.001**	−5.89 to 9.75
Type of Surgery, n			0.753	
MVR	7	8		
AVR	20	25		
MVR + AVR	14	17		
TVP	1	0		
Hct during CPB (%)	27.75 ± 2.37	27.10 ± 2.03	0.162	−0.26 to 1.56
Hct at the end of surgery (%)	29.83 ± 3.92	29.20 ± 3.55	0.429	−0.92 to 2.18
Red-cell transfusions (u)	1.11 ± 1.73	1.27 ± 1.94	0.677	−0.95 to 0.62
Plasma transfusions (ml)	140.95 ± 232.55	185.80 ± 285.72	0.417	−154.14 to 64.44
Fluid balance (ml)	1754.76 ± 831.75	1650.00 ± 777.82	0.535	−229.08 to 438.60
Urine output (ml)	694.52 ± 718.03	662.50 ± 480.24	0.799	−217.62 to 281.671

Continuous data are presented as mean ± standard deviation; HPG, high-pressure group; LPG, low-pressure group; CI, confidence interval; CPB, cardiopulmonary bypass; LVEF, left ventricular ejection fraction; AP, arterial pressure; AVR, aortic valve replacement; MVR, mitral valve replacement; TVP, tricuspid valvuloplasty; Hct, hematocrit.

**Table 3:** Postoperative data.

	HP (*n* = 42)	LPG (*n* = 50)	*p* value	95%CI
AKI, *n* (%)	17 (40.48%)	13 (26%)	0.140	
Delta Hct (%)	−9.33 ± 4.85	−10.63 ± 5.74	0.252	−0.93 to 3.52
Delta sCr (*μ*mol/L)	17.01 ± 21.74	12.27 ± 21.62	0.298	−4.27 to 13.76
Delta eGFR mL/ (min·1.73 m^2^)	−11.66 ± 17.46	−8.92 ± 21.02	0.504	−10.84 to 5.36
Extubation time (h)	17.91 ± 7.60	18.35 ± 13.89	0.855	−5.21 to 4.33
Length of ICU (h)	40.04 ± 37.61	35.07 ± 25.84	0.457	−8.24 to 18.17
Postoperative hospital stay (day)	13.05 ± 5.46	13.06 ± 5.30	0.991	−2.24 to 2.22

Percentages are given as total within group; continuous data are presented as mean ± standard deviation; HPG, high-pressure group; LPG, low-pressure group; CI, confidence interval; AKI, acute kidney injury; Hct, hematocrit; sCr, serum creatinine; eGFR, estimated glomerular filtration rate; ICU, intensive care unit.

**Table 4:** Subanalysis of risk factors associated with postoperative AKI.

	AKI (*n* = 30)	NO-AKI (*n* = 62)	*p* value	95% CI
Gender			0.034	
Male, *n* (%)	24 (80.00)	29 (46.77)		
Female, *n* (%)	6 (20.00)	33 (53.23)		
Age (year)	70.33 ± 4.69	70.55 ± 4.77	0.839	−2.31to 1.88
BMI (kg/m^2^)	23.64 ± 2.90	22.89 ± 3.82	0.342	−2.32 to 0.81
Hypertension (n)	13 (43.33%)	25 (40.32%)	0.860	
Hemoglobin (g/L)	127.03 ± 16.04	126.40 ± 17.30	0.867	−6.84 to 8.10
Hct (%)	38.30 ± 4.64	38.67 ± 5.02	0.740	−2.53 to 1.80
Preoperative sCr (*μ*mol/L)	84.67 ± 14.97	80.68 ± 16.63	0.269	−3.13 to 11.10
eGFR mL/ (min·1.73 m^2^)	81.81 ± 15,74	83.60 ± 18.61	0.650	−9.64 to 6.04
Preoperative LVEF (%)	59.73 ± 8.71	59.76 ± 8.04	0.989	−3.68 to 3.63
EuroSCORE II	3.33 ± 1.27	3.69 ± 1.17	0.181	−0.17 to 0.89
Aortic cross-clamp time (min)	106.80 ± 39.43	101.06 ± 42.13	0.534	−12.51 to 23.98
CPB time (min)	154.53 ± 49.44	138.37 ± 52.31	0.161	−6.55 to 38.88
Operation time (min)	302.23 ± 74.76	280.17 ± 100.20	0.288	−18.92 to 63.06
AP (mmHg)	65.68 ± 4.43	64.14 ± 6.80	0.209	−0.86 to 3.88
Delta Hct (%)	−13.57 ± 5.73	−12.07 ± 5.46	0.994	−2.39 to 2.37
Delta sCr (*μ*mol/L)	37.87 ± 17.15	3.09 ± 12.69	<0.001	−41.08 to 28.46
Delta eGFR mL/ (min·1.73 m^2^)	−28.17 ± 12.72	−1.46 ± 15.79	<0.001	−20.14 to 33.28
Red-cell Transfusions, n			0.304	
Yes, *n* (%)	15 (50)	24 (38.71)		
No, *n* (%)	15 (50)	38 (61.29)		
Plasma Transfusions, n			0.133	
Yes, *n* (%)	14 (46.67)	19 (30.65)		
No, *n* (%)	16 (53.33)	43 (69.35)		
Extubation time (h)	22.34 ± 17.12	16.11 ± 6.45	0.062	−0.34 to 12.81
Length of ICU (h)	47.27 ± 38.01	32.53 ± 27.16	0.064	−0.88 to 30.36
Postoperative hospital stay (day)	14.27 ± 6.64	12.47 ± 4.52	0.131	−4.14 to 0.55

Percentages are given as total within group; continuous data are presented as mean ± SD; AKI, acute kidney injury; CI, confidence interval; BMI, body mass index; Hct, hematocrit; sCr, serum creatinine; eGFR, estimated glomerular filtration rate; LVEF, left ventricular ejection fraction; CPB, cardiopulmonary bypass; AP, arterial pressure; ICU, intensive care unit.

## Data Availability

The data that support the findings of this study are available on request from the corresponding author. The data are not publicly available because of privacy or ethical restrictions.
